# Trial Watch - bispecific T cell engagers and higher-order multispecific immunotherapeutics

**DOI:** 10.1080/2162402X.2026.2632421

**Published:** 2026-02-16

**Authors:** Enfu Xue, Xiaolian Deng, Flora Doffe, Guido Kroemer, Oliver Kepp

**Affiliations:** a Université Paris Cité, Sorbonne Université, Inserm, Centre de Recherche des Cordeliers, Équipe labellisée par la Ligue contre le cancer, Institut Universitaire de France, Paris, France; b Université Paris-Saclay, INSERM US23/CNRS UAR 3655, Metabolomics and Cell Biology Platforms, Gustave Roussy, Villejuif, France; c Institut du Cancer Paris CARPEM, Department of Biology, Hôpital Européen Georges Pompidou, AP-HP, Paris, France

**Keywords:** Antibody engineering, bispecific and multispecific antibodies, cancer immunotherapy

## Abstract

Over the past decades, cancer immunotherapy has evolved into clinical practice, with bispecific T cell engagers (TCEs) and other higher-order multispecific immunotherapeutics emerging as approaches for precision immune modulation. These engineered antibodies redirect immune effector cells toward tumor targets, thereby eliciting coordinated antitumor immune responses. While substantial clinical success has been achieved in hematologic malignancies, major challenges, such as limited activity in solid tumors, continue to constrain broader application. In this Trial Watch, we summarize recent preclinical advances in the design and optimization of TCEs and multispecific immune engager platforms together with available clinical trial data. We further examine key pharmacokinetic, safety, resistance, and manufacturing considerations in immune engager development. Collectively, these findings highlight the increasing versatility of immune cell-redirecting immunotherapies and their pivotal role in shaping the next generation of precision cancer therapy.

## Introduction

**Figure 1.**
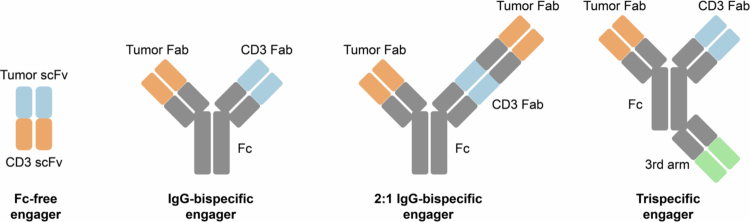
Schematic representation of molecular formats of bispecific and multispecific immune cell engagers. (A) Non-IgG-like, fragment-based bispecific antibodies such as BiTE® molecules consist of tandem single-chain variable fragments (scFvs) and lack an Fc region, resulting in compact size but short serum half-life. (B) IgG-like bispecific T cell engagers retain an Fc domain, which is frequently silenced (e.g. IgG4 backbone or Fc-engineering mutations) to prevent Fc-mediated effector functions while preserving extended pharmacokinetics. (C) Trivalent “2 + 1” formats represent bispecific antibodies with bivalent tumor antigen binding and monovalent CD3 engagement, enhancing tumor avidity without constituting true trispecific constructs. (D) Trispecific immune engagers incorporate a third functional specificity, such as albumin binding for half-life extension or costimulatory receptor engagement, enabling increased functional versatility at the expense of greater molecular complexity.

Cancer immunotherapy has transformed the landscape of oncology by harnessing the immune system to recognize and eradicate malignant cells.^1^ Over the past years, the field has evolved from broad immunostimulatory approaches, such as cytokine therapies and immune checkpoint inhibitors that augment existing MHC-dependent responses, to more precise and targeted strategies.^2^ Among the most promising advances are immune cell engagers, bioengineered antibody-based molecules that redirect immune effector cells toward tumors through direct cellular bridging, thereby enabling selective antitumor activity.^3–5^ T cell engagers (TCEs) represent the most clinically advanced subclass of immune cell engagers, distinguished by their ability to establish MHC-independent interactions between T cells and tumor cells through parallel recognition of tumor-associated antigens and T cell surface receptors.^6^ This interplay promotes the formation of functional immunological synapses and triggers cytotoxic effector programs within engaged T cells.^7^ By directly coupling the T cell receptor complex to tumor antigens, TCEs can circumvent tumor immune evasion driven by MHC downregulation and entirely bypass the requirement for conventional antigen presentation-dependent immune responses.^8^^,^^9^ Early clinical validation of this principle was achieved through the development of bispecific T cell engagers (BiTEs®) employing a single-chain variable fragment (scFv) format originally pioneered by Amgen. This approach, however, was constrained by short serum half-life, ultimately motivating a shift toward IgG-like bispecific antibodies that incorporate Fc regions to improve serum persistence and manufacturability. Accordingly, the majority of clinically advanced and approved TCEs now employ IgG-like bispecific formats. Building on these principles, the field is rapidly expanding beyond classical formats toward even more sophisticated approaches, including multispecific platforms (Figure 1).^10^ Next-generation immune cell engager designs incorporate additional binding domains to also engage NK cells, macrophages, and dendritic cells, fostering coordinated, multi-arm immune responses.^4,11–13^ Moreover, trispecific antibodies can synergize complementary activation pathways while integrating added functionalities such as cytokine delivery or checkpoint blockade within a single therapeutic molecule.^5^^,^^14^ This progression marks a transition from simple immune redirection to more comprehensive immune orchestration, with the potential to mitigate tumor-associated immunosuppression and resistance.^8^

In this Trial Watch, we address several key questions that currently shape the development of immune cell engagers: how molecular design choices influence pharmacokinetics, safety, and efficacy; which translational barriers limit activity, particularly in solid tumors; how emerging multispecific formats and conditional activation strategies aim to overcome resistance mechanisms; and how recent clinical experience informs future therapeutic directions. By integrating preclinical insights with clinical advances, we aim to provide a coherent framework for understanding the current landscape and future potential of T cell and immune cell engager-based therapies.

### Current molecular design and engineering strategies

T cell engagers encompass a broad and structurally diverse class of molecules that can be divided into two major categories based on the presence or absence of an Fc region.^15^ Non-IgG-like, fragment-based bispecific TCEs such as tandem scFv BiTE® molecules as well as alternative platforms, such as dual-affinity re-targeting (DART) molecules with stabilized heterodimeric scFv assemblies, were developed to enable compact size and rapid tissue penetration.^16^^,^^17^ One scFv binds a tumor-associated antigen, while the other targets CD3, an invariant component of the T cell receptor complex.^18^ The absence of an Fc region prevents engagement of Fc receptor-bearing cells such as macrophages, neutrophils, and natural killer cells, thereby limiting nonspecific immune activation by fragment-based bispecific TCEs.^17^^,^^19^ In contrast, IgG-like bispecific antibodies, which retain an Fc region, face distinct molecular engineering challenges related to heavy- and light-chain assembly as co-expression of distinct polypeptide chains can generate multiple mispaired species, necessitating specialized solutions to enforce heterodimer formation.^20^ To address this, strategies such as knobs-into-holes mutations and electrostatic steering have been developed to promote correct heavy-chain pairing.^21^^,^^22^ Additional platforms, including XmAb technology, further optimize heterodimerization by engineering differential isoelectric points between CH3 domains, enabling high heterodimer yields with minimal compromise to thermostability.^23^ Light-chain mispairing can be mitigated through approaches such as CrossMab domain crossover or common light-chain usage, enabling reliable large-scale production of IgG-like bispecific antibodies and facilitating their clinical translation.^20^^,^^24^^,^^25^ To further improve safety while preserving extended serum half-life, Fc regions are frequently silenced through the use of IgG4 backbones or Fc-engineering mutations that abrogate Fcγ receptor and complement binding, thereby preventing Fc-mediated antibody-dependent cell-mediated cytotoxicity (ADCC) and complement-dependent cytotoxicity (CDC).

The progression toward trispecific antibody engineering introduces further molecular and functional complexity, requiring integration of additional binding domains while maintaining structural integrity and therapeutic activity.^26^ Importantly, “2 + 1” formats, such as glofitamab, are trivalent bispecific antibodies that retain two antigen-binding arms for the tumor target and a single CD3-binding arm. In contrast, trispecific antibodies incorporate a third, distinct functional specificity, such as costimulatory engagement or albumin binding. Representative trispecific platforms include TriTAC molecules, which combine tumor targeting, CD3 engagement, and albumin binding to extend serum half-life.^14^ Advanced technologies such as BEAT and COBRA allow precise control of domain positioning, but increased molecular complexity also heightens risks of aggregation and misfolding, necessitating dedicated linker and stability engineering.^23^^,^^27^^,^^28^ Computational modeling and machine learning approaches are increasingly applied to navigate this expanding design space and optimize binding geometry, developability, and manufacturability.^29^^,^^30^

Beyond structural considerations, pharmacokinetic engineering is critical for clinical translation. Fusion to Fc domains, incorporation of albumin-binding motifs, or PEGylation are commonly employed to extend serum half-life and improve tissue exposure.^31^^,^^32^ Safety optimization further shapes molecular design, as high-affinity CD3 engagement can provoke severe cytokine release; accordingly, next-generation constructs increasingly rely on affinity tuning, split CD3 domains, or steric masking to balance cytotoxic potency with tolerability.^33^ Antigen selection is similarly integrated at the design stage, with preference for targets displaying high tumor specificity and sufficient density. Dual-antigen strategies are being explored to further enhance tumor selectivity and reduce on-target/off-tumor toxicity.^34^^,^^35^ At the clinical level, performance of TCEs is constrained by immune-mediated toxicity. Even with extended serum persistence, effective tissue exposure must be balanced against excessive immune activation. Consequently, the management of treatment-limiting toxicities, including cytokine release syndrome (CRS), immune effector cell-associated neurotoxicity syndrome (ICANS), and on-target/off-tumor effects, plays a central role in defining dosing regimens, therapeutic windows, and clinical applicability. Moreover, antigen selection and tumor heterogeneity remain central determinants of both efficacy and safety, with ideal antibody targets exhibiting high tumor specificity, sufficient surface density to trigger T cell activation, and minimal expression in normal tissues.

### Preclinical evaluation and translational insights

Preclinical development of bispecific and multispecific TCEs is primarily aimed at addressing three interrelated translational challenges: (i) establishing antitumor efficacy across heterogeneous targets, (ii) defining safety liabilities linked to T cell overactivation, and (iii) understanding resistance mechanisms that limit durability of response. A diverse set of experimental models has been deployed to interrogate these questions in a stepwise manner and guide clinical translation.

Efficacy assessment is commonly performed using syngeneic, xenograft, and humanized mouse models to evaluate dose-response relationships, antigen dependence, and immune cell recruitment.^36^^,^^37^ Humanized PBMC-reconstituted mice and patient-derived xenografts have been particularly informative for evaluating clinically relevant candidates, such as DLL3 × CD3 and EGFR × CD3 constructs, where enhanced CD8⁺ T cell infiltration and sustained tumor regression correlate with antigen density and target accessibility.^38^^,^^39^ These models have also highlighted the importance of molecular design parameters, including valency and Fc engineering, in shaping in vivo potency.^40^^,^^41^

Safety profiling represents a second critical translational pillar. Preclinical models are used to characterize cytokine release, T cell activation thresholds, and off-target immune engagement. Reduced CD3 affinity, split CD3 designs, and conditional activation strategies have consistently demonstrated preserved antitumor efficacy with attenuated systemic cytokine release in vivo, supporting their clinical relevance for improving therapeutic windows.^33^ Although murine models incompletely recapitulate human cytokine biology, comparative studies across formats remain valuable for ranking relative safety risk prior to first-in-human studies. Moreover, resistance mechanisms are increasingly interrogated in preclinical systems designed to mimic clinical relapse. Chronic exposure models revealed induction of T cell exhaustion, marked by upregulation of inhibitory receptors such as PD-1, TIM-3, and LAG-3, alongside reduced proliferative capacity. In parallel, myeloid-derived suppressor cells and tumor-associated macrophages can blunt T cell cytotoxicity, particularly in solid tumor microenvironments.^8^ Antigen heterogeneity and low target density further enable immune escape, providing a strong rationale for dual-antigen targeting and higher-order multispecific designs. Building on these insights, trispecific antibodies have emerged as a translational strategy to address limitations identified in bispecific platforms. Preclinical models demonstrated that trispecific constructs enhance tumor selectivity, reduce antigen escape, and sustain immune engagement by simultaneously targeting multiple tumor antigens or integrating immune-modulatory functions.^42^^,^^43^ Conditionally active trispecific antibodies, such as protease-activated formats, further improve tumor restriction in solid tumor xenografts, underscoring the value of microenvironment-aware design.^44^ Complementary studies using trispecific NK cell-engaging constructs reinforce the principle that multispecificity can overcome target heterogeneity and resistance mechanisms in settings where mono- or bispecific approaches fail.^45^

Collectively, these preclinical studies establish a model-driven translational framework in which efficacy, safety, and resistance are interrogated in parallel. The availability of syngeneic, humanized, and microenvironment-informed models enables rational optimization of multispecific T cell engagers and provides a mechanistic foundation for their continued clinical advancement.

### Clinical development and therapeutic impact

The clinical development of T cell engagers has progressed rapidly from early proof-of-principle to broadly adopted, disease-specific immunotherapies. Blinatumomab, the first FDA-approved BiTE® TCE, established T cell redirection as a viable therapeutic strategy in B-cell malignancies. Approved in 2014 for relapsed or refractory B-cell precursor acute lymphoblastic leukemia (B-ALL), the pivotal TOWER trial demonstrated superior overall survival compared with standard chemotherapy, with subsequent expansion to MRD-positive disease supported by high MRD clearance rates following single-cycle treatment.^46–48^ Phase I/II studies in relapsed or refractory B-cell non-Hodgkin lymphomas, including DLBCL, showed durable responses in a subset of patients.^49–51^ Blinatumomab has also been explored as consolidation therapy in high-risk DLBCL and in investigational settings beyond oncology.^52^^,^^53^ Together, these findings validated CD3-mediated cytotoxic redirection and informed subsequent T cell engager development. The trifunctional antibody catumaxomab provided clinical validation of multi-immune-cell engagement in EpCAM-positive carcinomas through simultaneous binding of tumor-associated EpCAM, CD3 on T cells, and Fcγ receptor-expressing accessory immune cells. Its clinical use required intraperitoneal administration and stepwise dosing to manage cytokine-mediated toxicities, and its architecture differs from the IgG-like bispecific formats that predominate in current TCE development. Nevertheless, catumaxomab represents an important precedent for coordinated immune effector recruitment translating into clinical benefits.^54^^,^^55^ Approved in Europe for the treatment of malignant ascites in EpCAM-positive carcinomas, catumaxomab was later withdrawn for commercial reasons. Nevertheless approval was renewed in February 2025.

Advances in antibody engineering and target selection subsequently drove the emergence of CD20 × CD3 bispecific antibodies as a dominant and clinically mature class in B-cell lymphomas. Mosunetuzumab, the first approved CD20-directed T cell engager, demonstrated durable responses with manageable CRS largely confined to early treatment cycles in relapsed or refractory follicular lymphoma, establishing CD20 as a robust and clinically versatile target. Building on this foundation, epcoritamab and glofitamab expanded the CD20 × CD3 therapeutic landscape through differentiated molecular architectures and treatment paradigms. Epcoritamab employs a DuoBody format enabling subcutaneous administration, which is associated with slower systemic absorption and generally lower peak cytokine exposure, whereas glofitamab incorporates a 2:1 CD20:CD3 configuration administered intravenously as a fixed-duration regimen with anti-CD20 pretreatment to mitigate cytokine release syndrome (CRS). Although both agents use step-up dosing strategies, intravenous delivery particularly in high-avidity 2:1 formats, has been associated with higher early cytokine peaks compared with subcutaneous administration. Both agents achieved high complete response rates in relapsed or refractory diffuse large B-cell lymphoma and received regulatory approval in 2023.^56–59^ Odronextamab further reinforced the clinical relevance of CD20 targeting, demonstrating substantial activity in heavily pretreated and post-CAR-T B-cell lymphomas and achieving regulatory approval based on durable responses.^60^^,^^61^ In multiple myeloma, T cell engager development rapidly converged on plasma-cell antigens. BCMA × CD3 bispecific antibodies, including teclistamab, elranatamab, and linvoseltamab, demonstrated robust efficacy in heavily pretreated, triple-class-refractory disease, achieving deep and durable responses that supported regulatory approval and validated BCMA as a clinically actionable target. Target diversification beyond BCMA was achieved with talquetamab, a GPRC5D × CD3 bispecific antibody that demonstrated clinically meaningful activity in patients refractory to BCMA-directed therapies, expanding the antigenic scope of T cell redirection in myeloma.^62^^,^^63^

Translation of T cell engager therapy into solid tumors has historically been challenging but recently achieved notable successes. Tebentafusp, a gp100 × CD3 bispecific TCR fusion protein, became the first approved T cell engager for solid tumors, significantly improving overall survival in HLA-A*02:01-positive patients with metastatic uveal melanoma and establishing proof-of-concept that T cell engagement can overcome key barriers in selected solid tumor contexts.^64^^,^^65^ Furthermore, tarlatamab, a DLL3 × CD3 bispecific antibody, recently achieved regulatory approval for small cell lung cancer, representing the first T cell engager approved for a neuroendocrine solid tumor.^66–68^

Collectively, these FDA- and/or EMA-approved T cell engagers illustrate the maturation of the field from early experimental formats to clinically optimized, disease-specific immunotherapies. Contemporary development now prioritizes validated targets, IgG-like formats with optimized pharmacokinetics, step-up dosing strategies, and outpatient-compatible administration, with CD20-directed therapies in B-cell lymphomas, BCMA- and GPRC5D-targeted agents in multiple myeloma, and emerging solid tumor indications defining the current clinical and translational standard of care (Table 1). Comparative analyzes indicate that platform design, route of administration, and CD3 affinity influence both efficacy and toxicity profiles. Both subcutaneous and intravenous T cell engagers can be administered using step-up dosing schedules to mitigate CRS; however, subcutaneous delivery is generally associated with slower absorption and lower peak cytokine levels, whereas intravenous administration, particularly for high-avidity formats such as 2:1 constructs, may result in more pronounced early cytokine exposure. These differences inform clinical decisions regarding dosing schedules, monitoring intensity, and combination strategies.^31^^,^^33^

**Table 1.** Approved T cell engagers.

Drug Name	Targets	Indication of first approval	Year of first approval	Design	Region of first approval
**Blinatumomab**	CD19 × CD3	Lymphoma	2014	scFvs-tandem	US
**Catumaxomab**	EpCAM × CD3	EpCAM^+^ carcinomas	2025	IgG2	Europe
**Elranatamab**	BCMA × CD3	Multiple myeloma	2023	IgG2Δak	US
**Epcoritamab**	CD20 × CD3	Diffuse large B-cell lymphoma	2023	IgG1	US
**Glofitamab**	CD20 × CD3	B-cell lymphomas	2023	2:1, IgG1	US
**Linvoseltamab**	BCMA × CD3	Multiple myeloma	2025	IgG4	US
**Mosunetuzumab**	CD20 × CD3	Follicular lymphoma	2022	IgG1	US
**Odronextamab**	CD20 × CD3	Non-Hodgkin lymphoma	2024	IgG4	Europe
**Talquetamab**	GPRC5D × CD3	Multiple myeloma	2023	IgG4	US
**Tarlatamab**	DLL3 × CD3	Extrapulmonary neuroendocrine tumors	2024	IgG1-Fc	US
**Tebentafusp**	gp100/HLA-A*02:01 × CD3	Metastatic uveal melanoma	2022	sTCR-scFv	US
**Teclistamab**	BCMA × CD3	Multiple myeloma	2022	IgG4	US

Data status as of January 2026.

The ongoing clinical development of canonical CD3-based T cell engagers spans a wide range of tumor antigens, molecular formats, and disease settings, reflecting continued optimization of direct T cell redirection strategies (Table 2). In solid tumors, the most advanced program is xaluritamig (AMG 509; STEAP1 × CD3), currently evaluated in the global, randomized phase III XALute trial (*NCT06691984*) in men with post-taxane metastatic castration-resistant prostate cancer, comparing xaluritamig with cabazitaxel or second-line androgen receptor-directed therapy.^69^ Additional solid-tumor programs include CLDN18.2 × CD3 constructs (such as AZD5863; *NCT06005493*) and GPC3 × CD3 antibodies (CM350; *NCT05263960*) in gastrointestinal malignancies, as well as DLL3 × CD3 engagement in small-cell lung cancer and neuroendocrine tumors using both conventional and albumin-binding formats (e.g. HPN328; *NCT04471727*) to enhance serum persistence. Conditionally activated CD3-based approaches targeting PSMA and EGFR (JANX007, *NCT05519449*; JANX008, *NCT05783622*) further illustrate efforts to improve safety and tumor selectivity while preserving cytotoxic potency.

**Table 2.** Selection of clinical trials assessing additional, canonical CD3-based T cell engagers.

Drug	Target	Indication	NCT	Phase	Status	Update
**AMG 509 (Xaluritamig)**	STEAP1 × CD3	Prostate cancer	NCT06691984	III	Recruiting	2026
**AZD5863**	CLDN18.2 × CD3	Solid tumors	NCT06005493	I/II	Recruiting	2026
**Cevostamab (BFCR4350A)**	FcRH5 × CD3	Multiple myeloma	NCT04910568	I	Active, not recruiting	2025
**CM336**	BCMA × CD3	Multiple myeloma	NCT05299424	I/II	Recruiting	2024
**CM350**	GPC3 x CD3	Advanced solid tumors	NCT05263960	I/II	Recruiting	2025
**HPN217**	BCMA × albumin × CD3	Multiple myeloma	NCT04184050	I	Active, not recruiting	2025
HPN328 (gocatamig; MK-6070) *	DLL3 × albumin × CD3	DLL3^+^ advanced cancers	NCT04471727	I/II	Active, not recruiting	2026
**IMA401**	MAGE-A4 × CD3	Solid tumors	NCT05359445	I	Recruiting	2025
**IMC-F106C**	PRAME × CD3	Solid tumors	NCT04262466	I/II	Recruiting	2025
**IMC-R117C**	PIWIL1 × CD3	Colorectal cancer; GI cancer	NCT06840119	I/II	Recruiting	2026
ISB 2001*	BCMA × CD38 × CD3	Relapsed/refractory multiple myeloma	NCT05862012	I	Recruiting	2025
JANX007**	PSMA × CD3	Prostate cancer	NCT05519449	I	Recruiting	2026
JANX008**	EGFR × CD3	EGFR^+^ solid tumors	NCT05783622	I	Recruiting	2025
**JNJ-88549968**	CALRmut × CD3	Myeloproliferative neoplasms	NCT06150157	I	Recruiting	2026
**LY4152199**	BAFF-R × CD3	B-cell malignancies	NCT07101328	I	Not yet recruiting	2026
**MGD024**	CD123 x CD3	Hematologic Malignancies	NCT05362773	I	Recruiting	2026
**ONO-4685**	PD-1 × CD3	T cell lymphoma	NCT05079282	I	Recruiting	2025
**Vibecotamab (XmAb14045)**	CD123 × CD3	Myelodysplastic syndromes, acute myeloid leukemia	NCT05285813	II	Active, not recruiting	2025

Gastrointestinal (GI). *Trispecific constructs are included where CD3 engagement is the principal effector mechanism. **Canonical CD3-based T cell engagers include constitutively active and conditionally activated (masked/prodrug) formats that mediate T cell redirection via CD3. Data status as of January 2026.

Hematologic malignancies remain a major focus of canonical CD3-based TCE development. Multiple BCMA × CD3 and FcRH5 × CD3 bispecific antibodies are under active investigation in multiple myeloma, including half-life-extended and trispecific designs (e.g. CM336, *NCT05299424*; HPN217, *NCT04184050*; ISB 2001, *NCT05862012*; cevo­stamab, *NCT04910568*). Additional programs extend canonical CD3 engagement to novel hematologic targets, including BAFF-R × CD3 (LY4152199; NCT07101328), CD123 × CD3 (MGD024, NCT05362773; vibecotamab, NCT05285813), and CALRmut × CD3 for CALR-mutated myeloproliferative neoplasms (JNJ-88549968; NCT06150157).^70^^,^^71^ Canonical CD3 engagement has also been extended to less conventional targets, such as PD-1 × CD3 bispecific antibodies for T cell lymphomas (ONO-4685; *NCT05079282*). In parallel, TCR-like antibody platforms that preserve CD3-mediated T cell redirection while enabling recognition of intracellular antigens presented on HLA molecules further broaden the accessible antigen landscape. Clinical candidates targeting PRAME, MAGE-A4, and PIWIL1 (IMC-F106C, *NCT04262466*; IMA401, *NCT05359445*; IMC-R117C, *NCT06840119*) exemplify this strategy. Collectively, these trials illustrate a pipeline dominated by early- and mid-stage clinical development, with a limited number of candidates already progressing toward late-stage validation.

Early clinical experience, particularly in solid tumors, has highlighted challenges related to tissue penetration, antigen heterogeneity, and immunosuppressive tumor microenvironments.^72^^,^^73^ Accordingly, contemporary development increasingly incorporates step-up dosing, conditional activation, and combination strategies, including checkpoint inhibition and costimulatory pathway modulation.^74^ Among trispecific constructs where CD3 engagement remains the principal effector mechanism, ISB 2001 (BCMA × CD38 × CD3) has demonstrated encouraging early activity with manageable cytokine release syndrome in a Phase 1 study (*NCT05862012*).^75^ In contrast, trispecific constructs integrating CD3 and CD28 costimulation, such as SAR442257 (*NCT04401020*), have encountered substantial safety liabilities, leading to trial termination despite modest efficacy.^76^^,^^77^

Together, the studies summarized in Table 2 highlight both the breadth and the current limitations of canonical CD3-based T cell engager development. While antigen diversity, molecular engineering, and conditional activation strategies continue to expand the therapeutic scope of this modality, durable late-stage success remains confined to a small number of highly optimized programs, underscoring the importance of target selection, format choice, and safety engineering for successful clinical translation.

### Remaining challenges and strategic solutions

#### Molecular design challenges

TCE antibody production faces intrinsic engineering challenges that limit manufacturability, primarily due to inefficient pairing of distinct heavy and light chains, which results in low yields, mispaired species, and increased purification burden.^78–80^ Compared with conventional monoclonal antibodies, bispecific formats also exhibit greater structural fragility, as incorporation of scFv domains, flexible linkers, and engineered interfaces reduces thermal stability and increases aggregation and fragmentation risk, particularly in CD3-engaging constructs.^81–83^ Notably, stability liabilities often emerge during scale-up rather than early discovery, making manufacturability a persistent translational bottleneck.^84^^,^^85^ These challenges are magnified in trispecific and higher-order multispecific antibodies, where additional domains, engineered disulfides, and non-native architectures increase risks of mispairing, misfolding, and aggregation.^86^^,^^87^ Variable-domain-only formats are particularly vulnerable due to the absence of stabilizing constant regions.^88^ As molecular complexity increases, manufacturing demands escalate, requiring specialized workflows and stringent quality control that directly impact scalability and commercial viability.^89^ To address these limitations, alternative architectures, including common light-chain designs, single-chain and domain-fused scaffolds, and modular multispecific platforms, have been developed to reduce combinatorial mispairing and improve yield, highlighting format selection as a key determinant of translational success.^10^^,^^90^

Immunogenicity further constrains development, as engineered variable regions, artificial linkers, and non-native interfaces can introduce neoepitopes that elicit anti-drug antibody responses, reducing exposure and efficacy under chronic dosing. Contemporary mitigation strategies emphasize extensive humanization, in silico T cell epitope prediction, and early integration of aggregation and linker optimization, with IgG-like scaffolds generally exhibiting lower immunogenic risk than highly engineered fragment-based formats.^81^

#### CD3 affinity optimization

Early T cell-engaging bispecific antibodies prioritized high-affinity CD3 binding to maximize T cell activation; however, clinical experience revealed that this approach frequently induces dose-limiting toxicities thereby constraining therapeutic windows.^91^^,^^92^ High-affinity CD3 engagement drives indiscriminate T cell activation and rapid systemic cytokine release, including TNF-*α*-mediated myeloid amplification, leading to fever, vascular instability, organ dysfunction, and limited dose escalation across multiple targets such as HER2, CLL-1, and CD38.^93–96^ These observations underscored the limitations of the high-affinity CD3 paradigm and the need to balance cytotoxic potency with systemic tolerability.^97^ Subsequent mechanistic and preclinical studies demonstrated that effective tumor killing does not require sustained high-affinity CD3 engagement or fully mature immunological synapse formation, supporting affinity-optimized designs that preserve efficacy while improving tolerability.^91^^,^^98^ However, optimal CD3 affinity remains highly context dependent, shaped by antigen density, tissue distribution, tumor microenvironment, and interspecies differences in CD3 kinetics, complicating dose prediction and translation.^99^^,^^100^ Accordingly, next-generation T cell engagers increasingly combine CD3 affinity tuning with safety-oriented engineering, including steric masking, prodrug formats, split-CD3 architectures, and dual-antigen logic gates, to confine activation to tumor cells and enable higher, therapeutically effective dosing while minimizing CRS and ICANS.^17^

#### Conditional activation and tumor-selective strategies

Solid tumors pose major challenges for bispecific antibody therapies due to physical and metabolic barriers within the tumor microenvironment that limit penetration, stability, and immune activity. Dense extracellular matrix, elevated interstitial pressure, and abnormal vasculature restrict antibody distribution and create heterogeneous drug exposure, often necessitating higher systemic dosing that still fails to achieve uniform intratumoral concentrations.^101–103^ Concurrently, immunosuppressive and destabilizing conditions, including hypoxia, acidosis, high lactate and adenosine levels, nutrient deprivation, and elevated extracellular ATP, impair T cell function and accelerate antibody degradation, further compromising efficacy.^104^^,^^105^ To address these limitations, conditional activation strategies have been developed to restrict bispecific antibody activity to tumor microenvironments. These include protease-cleavable masking, pH- or ATP-responsive unmasking, and conditional assembly systems that activate only in the presence of tumor-associated biochemical cues.^106^^,^^107^ However, tumor heterogeneity introduces variability in activating conditions across patients and lesions, and activation kinetics must be carefully balanced to avoid loss of efficacy or tumor selectivity.^108^^,^^109^ Despite these challenges, tumor-restricted activation strategies, such as dual-masked prodrugs and multi-input logic-gated constructs, are increasingly viewed as essential for expanding the therapeutic window and minimizing systemic toxicity in solid tumor settings.^110^

#### Platforms beyond canonical CD3-based T cell engagement

Clinical trials assessing immune engager platforms beyond canonical CD3-based T cell redirection illustrate diversification of both effector-cell recruitment and activation logic. Costimulatory and checkpoint-integrating constructs, such as the PSMA × CD28 bispecific antibody REGN5678, the CD38 × CD28 × CD3 trispecific antibody SAR442257, the OX40 × 4-1BB agonist FS120, and the PD-1 × CTLA-4 bispecific lorigerlimab (MGD019), aim to modulate T cell activity through noncanonical signaling rather than direct cytotoxic redirection. Alternative effector-cell recruitment strategies are represented by NK-cell-directed platforms, including the CD16 × IL-15 × CD33 trispecific killer engager GTB-3650, and by γδ T cell-engaging constructs such as PF-08046052, which exploit innate-like cytotoxic responses in solid tumors. Several nonclassical CD3-containing architectures are also under evaluation, including avidity-engineered trispecific antibodies (CBA-1535), nanobody-based constructs targeting PD-L1 and HLA-G (SOA101), and atypical CD3 engagers with broader immune engagement profiles (TNB-486). TCR-based platforms further extend immune engagement to intracellular antigens, exemplified by the PRAME/HLA-A*02:01-directed ImmTAC IMC-P115C. Finally, emerging delivery technologies, such as the mRNA-encoded CD19 × CD3 engager ABO2203, highlight ongoing efforts to improve controllability and safety through transient, programmable immune activation. Collectively, these studies demonstrate how noncanonical immune engager platforms seek to expand therapeutic scope while addressing safety and selectivity limitations inherent to classical CD3-only T cell engagement (Table 3).

**Table 3.** Selection of clinical trials assessing safety and efficacy of platforms beyond canonical CD3-based T cell engagement.

Drug	Target	Indication	NCT	Phase	Status	Effector cell
**ABO2203**	CD19 × CD3 (mRNA)	Relapsed or refractory B-cell non-Hodgkin lymphoma	NCT07072169	I	Recruiting	T cells
**CBA-1535**	5T4 × CD3 × 5T4	Advanced solid tumors	NCT07016997	I	Recruiting	T cells
**FS120**	OX40 × 4-1BB	Solid tumors	NCT04648202	I	Active, not recruiting	T cells + innate cell
**GTB-3650 (TriKE)**	CD16 × IL-15 × CD33	Myelodysplastic syndromes and refractory/relapsed acute myeloid leukemia	NCT06594445	I	Recruiting	NK cells
**IMC-P115C**	PRAME/HLA-A*02:01 × CD3	PRAME^+^ solid tumors	NCT07156136	I	Recruiting	T cells
**MGD019 (Lorigerlimab)**	PD-1 × CTLA-4	Cervical cancer	NCT05475171	II	Recruiting	Myeloid cells + T cells
**PF-08046052/SGN-EGFRd2**	EGFR × γδ TCR	Advanced solid tumors	NCT05983133	I	Active, not recruiting	γδ T cells
**REGN5678** **(Nezastomig)**	PSMA × CD28	Prostate cancer	NCT03972657	I/II	Recruiting	T cells
**SOA101**	PD-L1 × HLA-G × CD3	Advanced solid tumors	NCT07055594	I/IIa	Recruiting	T cells
**TNB-486 (AZD486, surovatamig)**	CD19 × CD3	Mature B-cell malignancies	NCT06564038	I/II	Recruiting	T cells + innate cells

Data status as of January 2026.

### Future perspectives

The therapeutic landscape of T cell engaging antibodies is undergoing profound transformation through the integration of computational design and advanced molecular engineering.^111^ Machine learning and artificial intelligence (AI) are increasingly applied to antibody discovery and design, enabling accelerated candidate generation, more accurate prediction of affinity and developability, and rational optimization of multispecific architectures.^112^ Structure-guided computational approaches have demonstrated particularly striking benefits, achieving up to 25-fold improvements in antibody efficacy compared with sequence-based optimization alone.^113^ In parallel, de novo AI-driven platforms can now generate humanized and optimized antibody candidates within markedly reduced timeframes, while simultaneously incorporating multiple design constraints such as affinity, specificity, and developability.^114^ AI-assisted design is particularly valuable for multispecific constructs, allowing simultaneous optimization of binding geometry, stability, pharmacokinetics, and manufacturability. Computational simulations also enable virtual screening for potential immunogenicity and aggregation-prone regions before experimental validation.

Clinically, development pipelines include a large number of multispecific molecules in active testing, with over 60 immune cell engagers specifically tailored for solid tumors, marking a substantial expansion beyond hematologic malignancies.^115^ Trispecific formats further enhance therapeutic potential by enabling concurrent targeting of multiple tumor-associated antigens to mitigate immune escape, or by engaging costimulatory receptors to augment effector function.^116^ Moreover, bispecific killer engagers (BiKEs) and trispecific killer engagers (TriKEs) belonging to the class of NK cell engagers (NKCEs) can redirect natural killer cell cytotoxicity, often through CD16 engagement. In addition, conditional activation technologies are advancing therapeutic indices, with next-generation dual-masked platforms demonstrating decreased off-target binding while preserving full potency upon tumor-specific activation.^117^ Integration with biomarker-driven patient stratification is increasingly central with high-resolution immunoprofiling and genomic characterization allowing the identification of patients with optimal antigen expression, immune infiltration, and minimal immunosuppressive signatures, thus enabling precision deployment of immune engagers. Moreover, the integration of immune cell engagers with cellular therapies is emerging as a transformative direction for next-generation immunotherapies.^118^ Clinical evidence underscores this trajectory, with a large number of CAR-T trials currently active and twelve immune cell engagers approved by regulatory agencies.^119^ T cell engagers offer immediate off-the-shelf availability compared to complex CAR-T manufacturing, while CAR-T therapies provide sustained cellular persistence that antibody-based engagers cannot achieve.^120^ Innovative convergence strategies include armored CAR-T cells engineered to secrete bispecific antibodies or T cell engagers, thereby combining durable cellular persistence with flexible antibody-mediated targeting.^121^^,^^122^ These approaches have been shown to modulate the tumor microenvironment, enhance CAR-T cell infiltration and persistence, and mitigate antigen escape, including in glioblastoma models.^123^^,^^124^ Collectively, such hybrid strategies seek to integrate the immediate cytotoxic activity of immune engagers with the sustained antitumor effects of cellular therapies. These approaches are being evaluated in both hematologic malignancies and solid tumors, with early data suggesting improved tumor penetration and reduced antigen escape.^118^

Immune engagers have been positioned within precision immunotherapy frameworks to enable biomarker-driven patient stratification and individualized therapeutic strategies.^6^ Recent studies have reported the application of multispecific antibodies beyond hematologic malignancies, extending to solid tumors through the concurrent targeting of tumor-associated antigens or immune checkpoint molecules to address resistance mechanisms.^125^ High-resolution genomic and immunoprofiling approaches now delineate neoantigen landscapes, immune infiltration characteristics, and resistance-related pathways that guide the rational design and clinical implementation of immune engager-based regimens.^126^ Emerging data indicate that immune cell-directed antibody formats are being systematically integrated into diverse therapeutic platforms, supported by expanding preclinical and clinical investigations across multiple oncological settings.^127^^,^^128^ Solid tumor translation remains a central challenge, with key hurdles including tumor heterogeneity, physical barriers, and immunosuppressive microenvironments. Strategies such as dual-antigen targeting, conditional activation, local delivery, and combination with checkpoint inhibitors or cytokine modulators are being actively explored to improve clinical outcomes.

In conclusion, the rapid convergence of computational design, molecular engineering, and clinical innovation has elevated immune cell engagers from niche therapeutic tools to foundational elements of precision immunotherapy.^35^^,^^129^ While significant challenges remain, the trajectory of current advances strongly suggests that immune engager-based therapies will play a defining role in the next era of cancer treatment and may reshape the broader landscape of immune-based interventions.^127^ Looking forward, continued integration of AI-guided design, multispecific optimization, biomarker-driven precision strategies, and combination with cellular therapies is likely to expand the therapeutic window, improve safety, and enable effective targeting of previously intractable solid tumors.

## Data Availability

Data sharing is not applicable to this article as no new data were created or analyzed in this study.
